# Adapt-cMolGPT: A Conditional Generative Pre-Trained Transformer with Adapter-Based Fine-Tuning for Target-Specific Molecular Generation

**DOI:** 10.3390/ijms25126641

**Published:** 2024-06-17

**Authors:** Soyoung Yoo, Junghyun Kim

**Affiliations:** 1 Department of Artificial Intelligence, Sejong University, Seoul 05006, Republic of Korea; yooso0731@sju.ac.kr; 2 Deep Learning Architecture Research Center, Sejong University, Seoul 05006, Republic of Korea

**Keywords:** small-molecule drug design, generative pre-trained transformer, SELFIES, fine-tuning

## Abstract

Small-molecule drug design aims to generate compounds that target specific proteins, playing a crucial role in the early stages of drug discovery. Recently, research has emerged that utilizes the GPT model, which has achieved significant success in various fields to generate molecular compounds. However, due to the persistent challenge of small datasets in the pharmaceutical field, there has been some degradation in the performance of generating target-specific compounds. To address this issue, we propose an enhanced target-specific drug generation model, Adapt-cMolGPT, which modifies molecular representation and optimizes the fine-tuning process. In particular, we introduce a new fine-tuning method that incorporates an adapter module into a pre-trained base model and alternates weight updates by sections. We evaluated the proposed model through multiple experiments and demonstrated performance improvements compared to previous models. In the experimental results, Adapt-cMolGPT generated a greater number of novel and valid compounds compared to other models, with these generated compounds exhibiting properties similar to those of real molecular data. These results indicate that our proposed method is highly effective in designing drugs targeting specific proteins.

## 1. Introduction

Drug design aims to generate new compounds with desired properties and/or activities. This is challenging as it requires finding a small number of molecules with desired properties within the vast chemical space, typically comprising $10^{33}$–$10^{80}$ potential molecules [1,2].

According to previous studies [3,4,5,6], it takes an average of over 10 years and USD 314 million∼USD 2.8 billion dollars for new drugs to go from research and development to market. Moreover, only 0.1% of drug candidates successfully progress from preclinical testing to human testing, and among them, only 1 in 5000 reaches the market [7]. Therefore, constructing candidates with promising drugs is one way to supplement the drug discovery pipeline [8]. A specific task known as small-molecule drug design aims to generate compounds that exhibit activity against specific protein targets, significantly narrowing the search space within the vast chemical space based on specific conditions.

The virtual screening (VS) [9] approach is used extensively to mine novel chemical entities from a predefined compound library based on molecular docking towards a known target structure or similarity to known active compounds, for assembling promising candidates. However, this approach has limitations as it only explores within the known compound library and does not represent the entire chemical space. To address the limitations of traditional methods, De Novo Drug Design (DNDD) has emerged, aiming to design novel chemical compounds from scratch that meet specific constraints [10,11]. This approach enables a broader exploration of chemical space by generating previously unseen molecules, which holds potential for innovative and improved therapeutic development.

With recent advancements in deep learning technology, various architectures such as recurrent neural network (RNN) [12,13], variational auto-encoder (VAE) [14], generative adversarial networks (GANs) [15,16], and reinforcement learning (RL) [17,18] models have been employed for the DNDD. Typically, deep learning-based drug generation algorithms use drug molecular data represented as strings, and involve steps to learn the grammar of these strings and to generate molecules with properties similar to those used in training. Recently, a new model known as the conditional generative pre-trained transformer (cMolGPT) [19], which is based on the generative pre-trained transformer (GPT) [20], has been proposed to generate active compounds targeting specific proteins. The generation process of this model is conditioned on specific targets using the embeddings of the target proteins as keys and values in the attention mechanism.

The target proteins for the model include EGFR, HTR1A, and S1PR1, and the molecular dataset used for model training is represented using the simplified molecular input line entry system (SMILES) [21]. The authors conducted model training in two phases: unsupervised pre-training and supervised fine-tuning. Initially, the model learns to generate drug-like compounds, and it is then fine-tuned specifically for target proteins. The model underwent comprehensive evaluation through various experiments in drug-like compound generation and target-specific active compound generation tasks. Specifically, the performance of the cMolGPT was verified using common quantitative metrics in the DNDD field, the chemical properties of generated molecules, and activity prediction. The cMolGPT demonstrated a superior performance in generating new virtual compounds compared to other models, producing molecules that resemble those in the training data. However, the specific data used in fine-tuning was extremely limited—only about 0.3% of the pre-training data—which resulted in the lower performance in generating target-specific compounds compared to drug-like compound generation without a target.

We focused on designing a model capable of generating virtual compounds with a high performance, addressing the performance degradation issue caused by the limitation of the dataset size. Firstly, to improve the efficiency of the proposed model, we change the molecular representation method from SMILES to SELF-referencing embedded strings (SELFIES) [22]. SELFIES is a method proposed to overcome the limitation of SMILES representation, such as its inability to efficiently handle rings, branches, and bonds between atoms. It expresses molecules in string form by utilizing new derivation rules that take into account chemical and syntactic meaning, thereby ensuring that the model only generates valid molecules. Secondly, we modify the overall fine-tuning process to enable the generation of compounds without significant performance degradation, even when using a small-scale, target-specific dataset. Specifically, we add a new adapter module to the existing model structure to learn high-dimensional features for generating specialized targets. Additionally, a newly designed fine-tuning training strategy is used to adjust the model’s parameters to detailed goals without performance degradation.

Through various performance experiments, we evaluate and analyzed our method from different perspectives, comparing the results with those of previous models [19] to confirm the performance improvement effect of our proposed method. As a result, the Adapt-cMolGPT was able to generate 100% valid molecules by modifying the molecular representation method. Moreover, the model demonstrated a superior performance in generating target-specific compounds compared to the comparative models, with the generated compounds exhibiting chemical properties similar to those of the real data while displaying high activity.

## 2. Results and Discussion

We evaluated the performance of our Adapt-cMolGPT in two tasks: (a) the capability of generating drug-like virtual compounds using a pre-trained model, and (b) the feasibility of generating target-specific active compounds using a fine-tuned model. In both tasks, we assessed the performance improvement effect of the proposed method in this paper by comparing it with the existing model, cMolGPT. In this case, we conducted the re-training and re-inference of the existing model to verify the results of the newly defined performance metric in this study.

### 2.1. Drug-like Compound Generation Results from Pre-Trained Model

To check the performance of the drug-like compound design, we employed a pre-trained model of our proposed method to generate 30,000 molecules and conduct various numerical performance evaluations and compound property analyses. First, we calculated three quantitative metrics commonly used in the DNDD field, including the fractions of syntactically and semantically valid (valid), unique (Unique@1k, Unique@10k), and novel (novel) molecules; these were presented in Table 1. Among these metrics, unique and novel were only calculated for valid molecules. Specifically, uniqueness was calculated for 1000 and 10,000 extracted valid molecules, respectively, labelled as Unique@1k and Unique@10k. As shown in Table 1, the Adapt-cMolGPT achieved a performance close to 1, which is the maximum performance, in all three metrics. Our model demonstrated performance improvements over cMolGPT, particularly in the valid and novel metrics. In other words, the Adapt-cMolGPT generated novel virtual compounds without duplicates even during the training, and all generated molecules were both syntactically and semantically valid.

**Figure 1:**
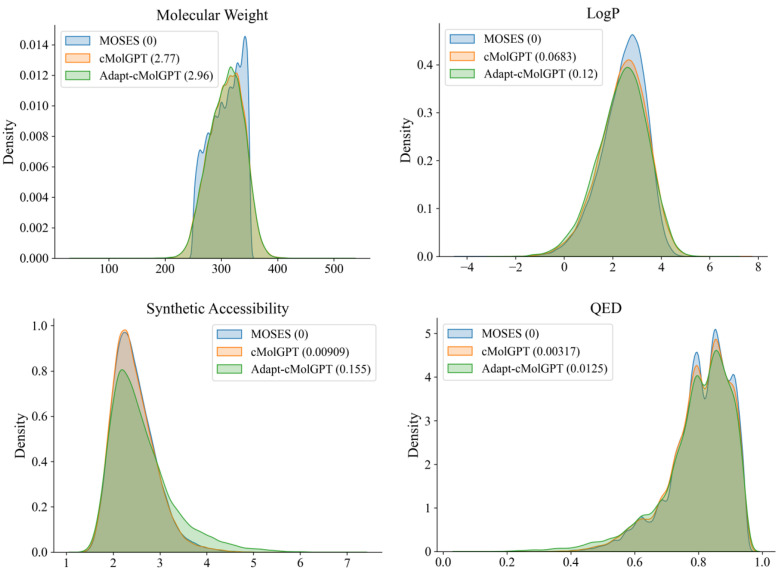
Distribution of chemical properties for data samples from the MOSES dataset and data samples generated by cMolGPT and Adapt-cMolGPT, respectively.

In addition, we investigated the distributions of the four molecular properties to verify whether these diverse and novel compounds generated by the Adapt-cMolGPT reflect the properties of the real drug molecules, as shown in Figure 1. In the figure, MOSES represents the test set of the real molecule dataset, with the numbers in parentheses indicating the 1-Wasserstein distance between the MOSES test set and the set of generated virtual molecules. The 1-Wasserstein distance measures the distance between two probability distributions, so indicating that the smaller this distance, the more closely our model generates compounds that resemble the corresponding chemical property of the real data. Our model had the closest distance of 0.0125 in terms of QED, and the farthest at 2.96 in terms of molecular weight. Overall, it has been observed that the distribution of the chemical properties of the data samples generated by the proposed Adapt-cMolGPT closely resembles that of the data samples from the MOSES dataset, which comprises real drug molecules. This shows the capability of the Adapt-cMolGPT to generate drug-like compounds that reflect the chemical properties of real molecules. In summary, our Adapt-cMolGPT was capable of generating diverse and valid drug-like compounds with the desired properties, even without a target protein.

### 2.2. Target-Specific Compound Generation Results from Fine-Tuned Model

To evaluate the performance of target-specific compound generation, we utilized a fine-tuned model of our proposed method, using EGFR, HTR1A, and S1PR1 protein as the targets. We sampled 30,000 compounds for each target, then calculated the fractions of the validity, uniqueness, and novelty of molecules, which are presented in Table 2. The performance was compared with cMolGPT and conditional RNN (denoted by cRNN), where cRNN is an RNN-based conditional model used for comparing the result of cMolGPT in previous study. It was also initially trained on the MOSES set and then fine-tuned on the target-specific set. The results indicate that the validity and novelty scores for all cases were at the maximum value of 1, surpassing those of the comparison models. Additionally, in terms of the uniqueness of 10k valid compounds, our model achieved values of 94%, 96%, and 93% for EGFR, HTR1A, and S1PR1, respectively, demonstrating its significant superiority over the comparison models. In summary, in the molecular generation task with specified targets, Adapt-cMolGPT generates valid compounds that have not been seen during the training and generation steps. Moreover, while cMolGPT experienced performance degradation in both validity and uniqueness metrics, dropping to approximately 0.9 and 0.86 from 1, respectively, our model showed only a slight decrease in uniqueness, which was less pronounced compared to that model.

**Figure 2:**
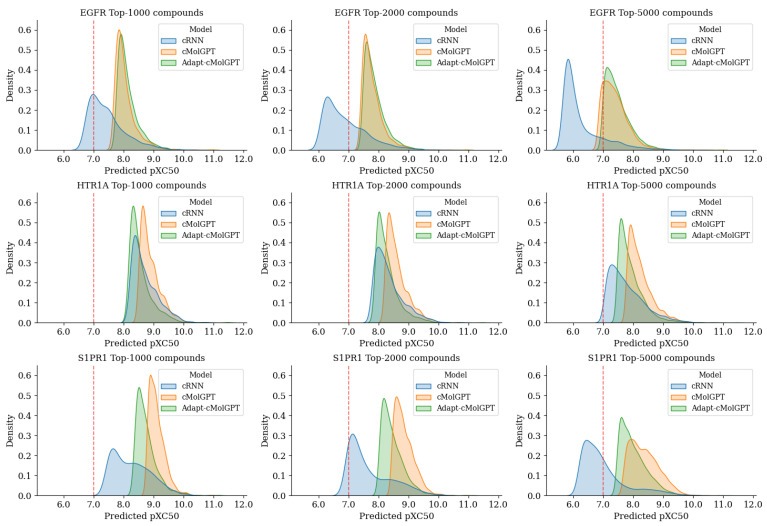
Distributions of predicted activity (pXC50) for the top 1000 (left), 2000 (middle), and 5000 (right) compounds from cRNN (blue), cMolGPT (orange), and the proposed Adapt-cMolGPT (green), for the EGFR (top), HTR1A (middle), and S1PR1 (bottom) targets.

To comprehensively assess the efficiency of compounds sampled by our Adapt-cMolGPT, we predicted their activity using the quantitative structure–activity relationship (QSAR) models for the generated valid compounds and plotted the distributions. The activity distribution depicts the top 1000/2000/5000 compounds based on predicted activity values, as shown in Figure 2. The activity value, pXC50, used in the evaluation, represents the degree of target response according to the dose of the therapeutic agent containing a specific compound, and the higher this value, the lower the required therapeutic dose. The pXC50 allows for categorizing the activity classes of each compound based on specific threshold values. According to previous research [23], the activity classes are typically categorized as ‘low’ when the predicted pXC50 value is less than 6, ‘middle’ when it is above 6 but less than 7, ‘high’ when it is above 7 but less than 8, and ‘ultra-high’ when it exceeds 8. The red dashed line in the figure represents the threshold for the ‘high’ class, and the results show that our model’s predicted activity values exceed this threshold for all three objectives. Specifically, our model generated significantly more compounds with high activity in the EGFR and S1PR1 proteins compared to cRNN. In nearly all cases, both cMolGPT and Adapt-cMolGPT produced compounds falling within the same activity class. In other words, our proposed Adapt-cMolGPT generates compounds with high activity against all three proteins, similarly to cMolGPT.

**Figure 3:**
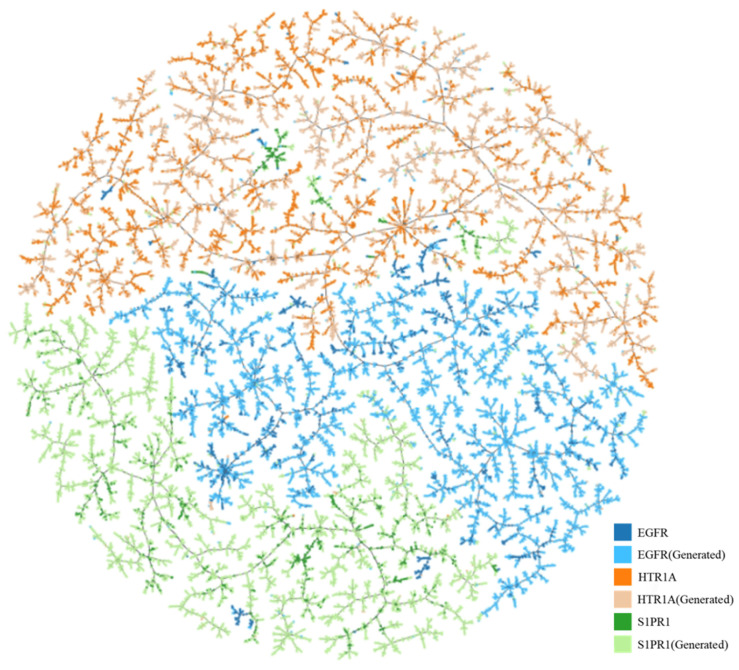
TMAP of the target-specific molecules generated by the proposed Adapt-cMolGPT (light colors), as well as the ground-truth target-specific molecules (dark colors).

Moreover, we conducted visualizations to analyze the positioning of the generated target-specific compounds within the chemical space, based on the hypothesis that molecules targeting the same protein would be densely clustered. For the visualization, we selected the top 5000 predicted active compounds among the generated valid compounds for each target protein. We computed the MinHash fingerprint vectors [24] for these molecules and then projected them onto a 2D space using the Tree MAP (TMAP) [25]. As shown in Figure 3, each node corresponds to a molecule, with light and dark colors representing the generated virtual compounds and real target-specific compounds, respectively. As anticipated, the generated molecules were closely positioned to the real molecules for each target, demonstrating that Adapt-cMolGPT can generate compounds similar to those in the training dataset.

Finally, we investigated whether our Adapt-cMolGPT generates compounds with physicochemical properties similar to those of real molecules. We evaluated five properties (molecular weight, TPSA, LogP, HBD, and HBA), quantitative estimate of drug-likeness (QED), and synthetic accessibility (SA) scores for the top 5000 generated active compounds. Here, QED is a score that reflects the underlying distribution of several molecular properties, such as the number of aromatic rings and rotatable bonds, as well as the five properties mentioned above. This score ranges from 0 to 1, with higher values indicating greater ‘drug-likeness’. SA is calculated as the sum of the fragment score and complexity penalty. This score ranges from 1 to 10, with higher values indicating more difficult synthesis. These properties are effectively used to predict whether the compounds possess the desired pharmacokinetic properties. Subsequently, we calculated the percentage of molecules that fell within the ‘good’ drug-like property ranges, as defined in the previous study [19], for each property and summarized these in Table 3. The results indicate that the compounds sampled by our method fell within the defined range for most properties, with nearly 80% exceeding this threshold across the three targets. Particularly, for the four physicochemical properties (TPSA, LogP, HBD, and HBA), nearly 90% of the compounds in almost all cases were within the ‘good’ property range. In summary, our Adapt-cMolGPT is capable of generating drug-like compounds for each target.

In conclusion, our model successfully generated entirely novel, target-specific compounds with 100% validity that were unseen during the training process. Through various analyses, we confirmed that the generated compounds exhibit similar properties for each target and are closely positioned within the chemical space. Consequently, our Adapt-cMolGPT demonstrates high performance in generating drug-like active compounds, even in target-specific drug design tasks.

## 3. Methods and Materials

### 3.1. Dataset

We sequentially conduct the pre-training and fine-tuning of the model for target-specific molecular generation. The dataset used in each stage is identical to the dataset used in the previous study [19]. We utilized the MOSES molecular dataset [26] for pre-training, which comprises 1,760,739 drug molecules extracted from the ZINC clean Lead Collection [27], divided into training and testing sets in a 9:1 ratio, consisting of 1,584,664 and 176,075 molecules, respectively.

For fine-tuning, we used the dataset that concatenates target protein and the corresponding molecular data [16]. Specifically, the target-specific dataset comprised 1381, 795, and 3585 molecules for the EGFR, S1PR1, and HTR1A proteins, respectively. Given the varying sizes of datasets for each target, we divided them into a 9:1 ratio and integrated them.

### 3.2. Molecular Representation Methods

To design molecules using computer-based models, each molecule needs to be represented as a string type. The most commonly used method for string-based molecular representation is SMILES [21]. SMILES represents molecules based on the principles of molecular graph theory; however, there have been issues with many representations being syntactically invalid and violating basic chemical rules. To solve this problem, several new representation methods have been proposed. Among these, SELFIES [22] aimed to fundamentally address the invalidity problem by redefining SMILES itself.

The SELFIES utilizes new derivative rules that consider chemical semantics and stores information about branch length and ring size, along with their identifiers, ensuring that syntactically and semantically valid molecules are generated with 100% robustness. Recently, a study [28] emerged, improving the performance in various tasks by utilizing the molecular data represented in SELFIES within Transformer-based models. In their previous paper, the authors demonstrated the benefits of using SELFIES strings for model training in the context of chemical language modeling, using experimental results. Inspired by these results, we adopted SELFIES as the representation for drug molecules to generate novel and valid molecules. Since the datasets used in the previous study were represented in SMILES, we underwent the process of converting them into SELFIES before inputting them into our model.

### 3.3. Generative Pre-Trained Transformer for Conditional Molecular Generation

The GPT [20] is a Transformer-based architecture and training procedure designed for natural language processing. It utilizes pre-trained knowledge to generate tokens for subsequent outputs, with training proceeding in a two-step process. Initially, the neural network model’s parameters are trained using unlabeled data. Subsequently, fine-tuning is conducted with labeled data to adapt to the target task. Specifically, GPT consists of layers composed of multi-head attention (MHA) and feed-forward network (FFN) blocks stacked together.

The MHA consists of *h* attention heads that extract relevant features from the input data in parallel. Each head of MHA calculates attention with query, key, and value denoted as $Q\in R^{T\times d_{model}},K\in R^{T\times d_{model}}$, and $V\in R^{T\times d_{model}}$, respectively, where *T* is the length of the input sequence and $d_{model}$ represents the embedding dimension of the model.

In this case, the query matrix is represented as $Q_{i}=Q{W_{i}}^{Q}$, the key matrix as $K_{i}=K{W_{i}}^{K}$, and the value matrix as $V_{i}=V{W_{i}}^{V}$, where *i* represents the index of attention heads, ranging from 1 to *h*. ${W_{i}}^{Q}\in R^{d_{model}\times d_{k}}$, ${W_{i}}^{K}\in R^{d_{model}\times d_{k}}$, and ${W_{i}}^{V}\in R^{d_{model}\times d_{v}}$ are learnable parameter matrices. Here, $d_{k}$ and $d_{v}$ denote the dimensions of the key and value, respectively. The attention formula for the *i*-th head is defined as follows:

(1) $$\begin{array}{c} Head_{i}=Attention\left(Q_{i},K_{i},V_{i}\right)\\ \;\;=softmax\left(\frac{Q_{i}{K_{i}}^{T}}{\sqrt{d_{k}}}\right)V_{i}, \end{array}$$

where ${\left(\cdot \right)}^{T}$ is the transpose operation.

The MHA concatenates single-head attention ${Head}_{i}\in R^{T\times d_{v}}$ in parallel with the number of heads *h* and then calculates it as follows:

(2) $$MultiHeadAttention\left(Q,K,V\right)=Concat\left(Head_{1},\ldots ,Head_{h}\right)W^{O},$$

where $W^{O}\in R^{hd_{v}\times d_{model}}$ is learnable parameter. In this study, we employ the embedding dimension $d_{model}=512$ and h=8 parallel attention heads. Additionally, we set the dimensions of key and value, denoted by $d_{k}=d_{v}=d_{model}/h=64$.

The FFN in each layer helps alleviate bottlenecks in embedding vector dimensions by first expanding and then compressing the dimensions of input features. Each block includes normalization, dropout, and residual connection processes. Ultimately, the features extracted through multiple layers are processed by the output layer to produce values tailored to the task at hand. The GPT has achieved state-of-the-art performance across various tasks and is widely utilized in various fields.

Building on the GPT architecture, cMolGPT is a specialized model designed to generate molecules for specific target proteins. It consists of three decoder blocks and an output layer, where the decoder blocks resemble those in GPT but include an additional layer of MHA. Notably, in the second MHA block, embedding data for the target proteins are fed into *Q* and *K*, facilitating specialized generation tailored to the targets. Training follows the same two-step process as GPT. In the pre-training stage, initial parameters are trained using unlabeled data, while in the fine-tuning stage, guided data specifying the target proteins are used to generate molecules tailored to the objective.

We adopt the structure of cMolGPT to generate target-specific molecules. The enhancements that make cMolGPT a more efficient model for designing target-specific molecules will be detailed in the subsequent sections.

### 3.4. Workflow for Training

**Figure 4:**
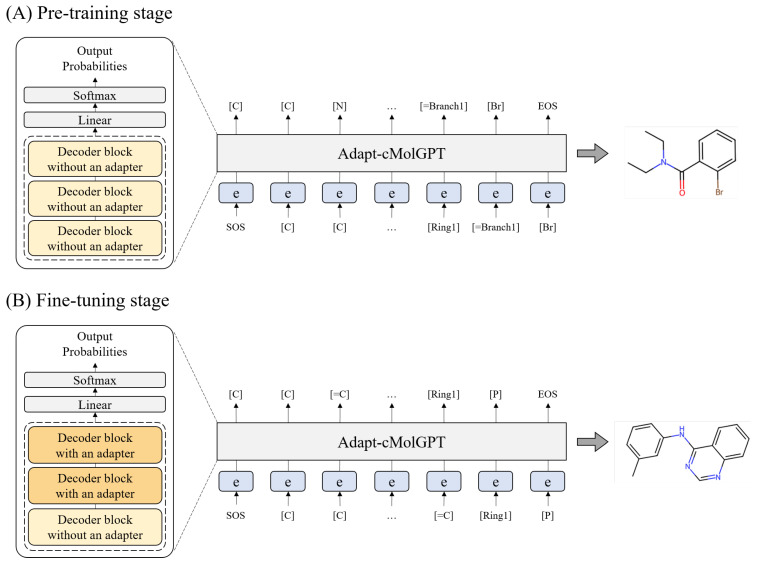
Workflow of adapt-cMolGPT.

As described earlier, the proposed Adapt-cMolGPT is designed to address two primary tasks: (1) generating diverse, valid drug-like compounds and (2) generating target-specific active compounds. To achieve this, we proceeded sequentially with the pre-training and fine-tuning stages, as illustrated in Figure 4. In the pre-training stage, we utilize a large-scale dataset of drug molecules, which lack specified targets, to train the model on the structure and grammar of SELFIES strings. Subsequently to this, the model is fine-tuned to generate compounds for specific target proteins, employing a smaller-scale dataset that includes specified targets.

In the fine-tuning stage, an adapter module is added to the pre-trained model architecture, and the method for updating the model’s weights is modified to enable the learning of crucial information required for target-specific generation. Accordingly, we define the decoder blocks used in the pre-training as ‘decoder blocks without an adapter’ and in the fine-tuning as ‘decoder blocks with an adapter’. The structure of Adapt-cMolGPT, as utilized in each training stage, will be detailed in the respective subsections.

All experiments were performed on a single Nvidia GeForce RTX 4090 Ti GPU, using Python 3.8.18 and PyTorch 2.2.0. To optimize the model’s weights, we used the Adam optimizer with a batch size of 512.

### 3.5. Pre-Training Process

**Figure 5:**
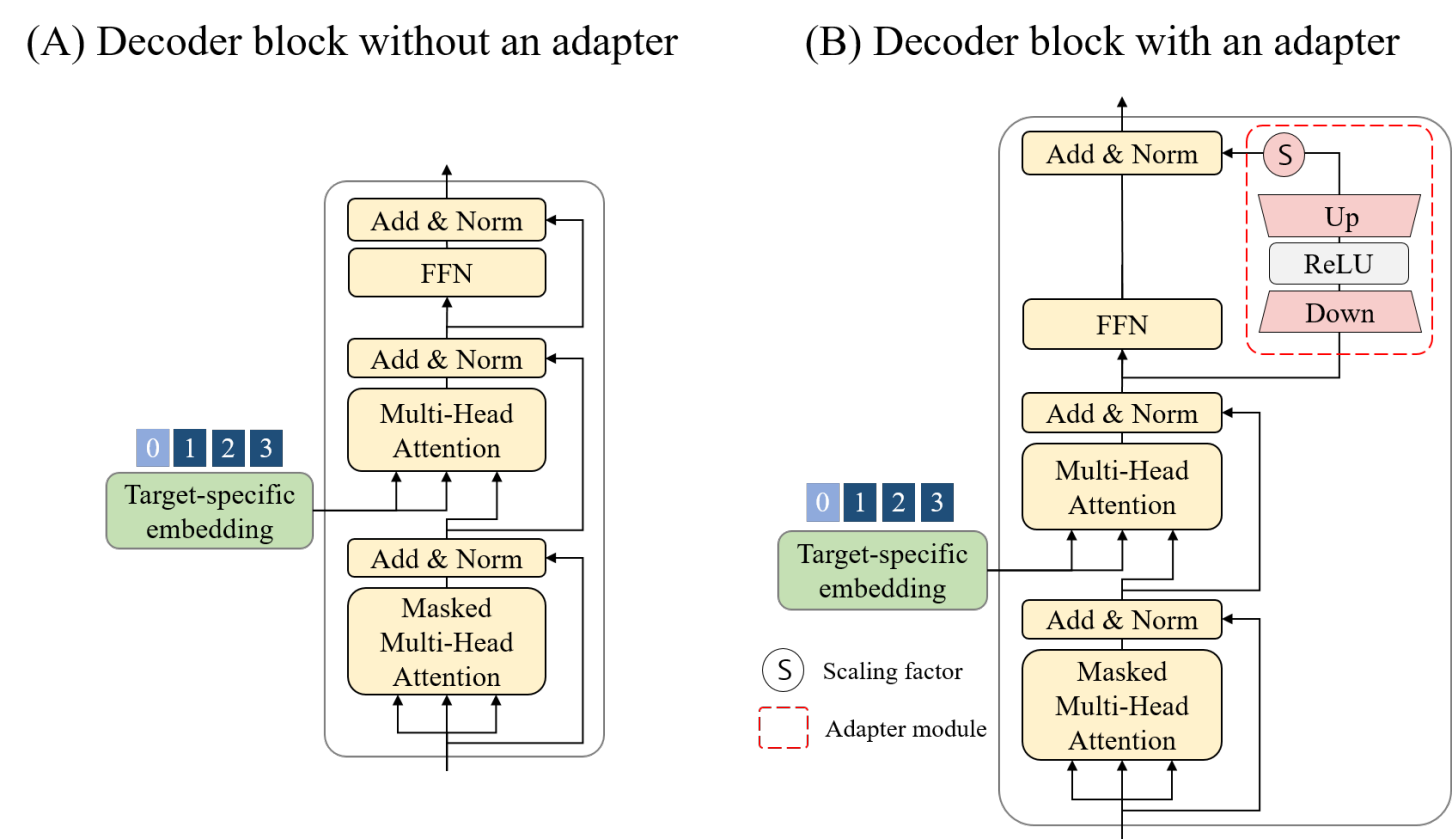
The architecture of decoder blocks in Adapt-cMolGPT.

In the pre-training of Adapt-cMolGPT, we utilized a model composed of three stacked decoder blocks with adapters, as shown in Figure 4A. This structure is identical to that of cMolGPT, and the architecture of the decoder block is illustrated in Figure 5A. The target protein embedding is input into the second MHA layer of the decoder block. During the pre-training process, the conditional embedding is initialized with zeros, because the task is to generate valid molecules without the target. Unlike previous approaches, we used molecular data represented in SELFIES and trained the model for 100 epochs. The learning rate began at 0.0001 and decreased by 0.95% per epoch.

### 3.6. Fine-Tuning Process

In the fine-tuning of our Adapt-cMolGPT, we modify the architecture of the pre-trained model and introduce a new training strategy. Specifically, we add an adapter module, designed for the efficient fine-tuning in previous research [29], to the decoder blocks of our pre-trained model. Additionally, we define and group the layers to be fine-tuned into two sets, and alternate the training of these groups sequentially.

The model used in the fine-tuning stage, as shown in Figure 4B, consists of a sequence of one existing decoder block without an adapter, as used in pre-training, and two newly designed decoder blocks, each with an adapter. As illustrated in Figure 5B, the adapter module is a trainable down- and up-projection bottleneck module, enabling the more efficient learning of specialized features for each target.

Fine-tuning involves additional training of the pre-trained model using a small-scale dataset for a specific task. Typically, full fine-tuning, which is widely used, involves re-training all parameters of the pre-trained model. This method shows high performance but incurs significant computational costs and the risk of forgetting previously learned information. To address these limitations, various fine-tuning methods [30,31,32] for selecting layers to fine-tune have been proposed.

Adapter fine-tuning [30], for instance, adds a new module, the adapter, to the pre-trained model structure, and only trains the parameters of the added adapter. This method drastically reduces the number of trainable parameters, lowering computational costs while achieving a performance similar to or even surpassing full fine-tuning. However, it may take longer to converge and pose the risk of overfitting.

**Figure 6:**
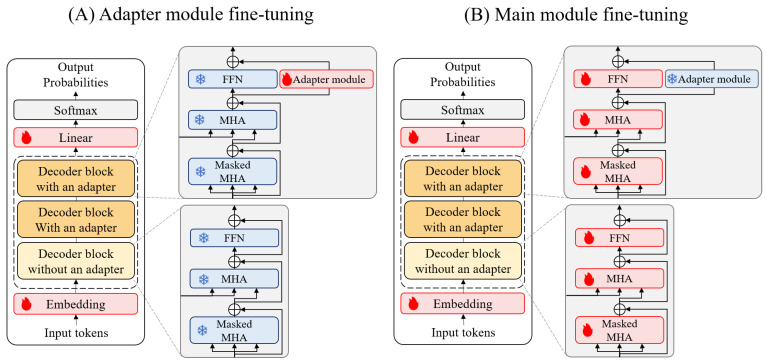
The architecture of two fine-tuning methods.

To reduce the computational complexity and prevent overfitting, we propose a training strategy that alternates training by dividing the layers to be fine-tuned. As shown in Figure 6, we organize the fine-tuning process into adapter module fine-tuning, where only the newly added adapter module in the decoder block is trained, and main module fine-tuning, where the adapter module is frozen, and the remaining layers are trained. The embedding layer and the linear layer are trained in each epoch. In the figure, the layers trained with each fine-tuning method are highlighted in red, while the frozen layers are marked in blue, clearly illustrating the distinct phases of our fine-tuning strategy. Through this strategy, our fine-tuning model can capture various aspects of the small-scale target-specific data. The adapter module fine-tuning and the main module fine-tuning are sequentially trained for 15 and 10 epochs, respectively. These two fine-tuning processes alternate.

During the fine-tuning process, the proteins EGFR, HTR1A, and S1PR1 are sequentially encoded as integers 1, 2, and 3, and their corresponding embedding vectors are then utilized as target-specific embeddings. In the experiment, the training was conducted for a maximum of 200 epochs, and early stopping was applied if the loss value of the validation set did not decrease for more than five epochs. In this case, the learning rate followed a cosine schedule, gradually increasing from 0 to 0.001 over the first 20 epochs, and then decreasing back to 0 by the 100th epoch, with this cycle repeating twice.

### 3.7. Generation Process

All drug generation models used in this study operate in an auto-regressive manner, which involves predicting the next molecular token at each step and using this token in subsequent steps. Specifically, the model predicts the probability of generating all molecular tokens in the vocabulary at each time step and determines the predicted token based on an appropriate sampling method. In cMolGPT, the authors used the top-*k* sampling method with *k* set to 30. We utilized temperature sampling with a temperature of 1.5 for more diverse molecule generation. Inference begins when the start token <SOS>, indicating the start of drug molecule generation, which is input into the model and ends when the end token <EOS> is output from the model.

### 3.8. ML-Based QSAR Model for Active Scoring

We evaluated the target-specific generation performance of our model using a regression-based QSAR model for each target. For this activity prediction, we utilized the model and data designed in the previous study [19]. According to this paper, the molecular dataset containing activity data for each target was extracted from ExCAPE-DB [33], comprising 5181, 6332, and 1400 molecules corresponding to the EGFR, HTR1A, and S1PR1 proteins, respectively. These authors trained a LightGBM [34] model using a total of 2533 molecular features, including a 2048-length FCFP6 fingerprint, a 166-length MACCSkeys, and 319 RDKit molecular descriptors, for activity prediction.

### 3.9. Performance Metrics

We initially evaluated the performance using three metrics (valid, unique, novel) across two tasks: one involving target proteins and the other without. These metrics are commonly used to evaluate a drug design model’s performance in DNDD, with detailed descriptions and formulas for each metric presented below. All three metrics indicate better a performance with higher values, with a maximum value of 1.

The valid metric represents the fraction of syntactically and semantically valid compounds within the generated compound set. In our experiments, we sampled 30,000 compounds using the generation models and extracted valid ones using the RDKit library in Python. The generated compound set *G* and the valid compound set *P* within *G* are defined as follows: $G=\left\{g_{1},\ldots ,g_{a}\right\}$, where *a* is the number of generated compounds. $P=\left\{p_{1},\ldots ,p_{b}\right\}$, where *b* is the number of valid compounds. Therefore, the valid metric is calculated as:

(3) $$Valid=\frac{n(P)}{n(G)},$$

where n(·) denotes the number of elements in the set.

The Unique metric represents the fraction of unique compounds in the generated valid compounds. We specified the size of the valid compound set used for uniqueness calculation as either 1k or 10k, defined as Unique@1k and Unique@10k, respectively. We defined the subset *S* and the unique compound set *U* from the valid compound set *P* as follows: $S=\left\{s_{1},\ldots ,s_{c}\right\}$, where S⊂P and *c* is 1k or 10k. $U=\left\{u_{1},\ldots ,u_{e}\right\}$, where *e* is the number of unique compounds after removing duplicates from the subset *S*. Now, the Unique metric is calculated as:

(4) $$Unique=\frac{n(U)}{n(S)}.$$

The higher the Unique value, the more diverse the generated compounds are without duplicates, indicating the effectiveness of the generation model. Typically, this metric can have lower values as the number of generated compounds increases, as there is a higher probability of duplicates. Therefore, the model showing high performance in unique@10k indicates its capability to generate a diverse set of valid compounds without duplicates.

In various prior drug generation studies, the above metrics have been commonly used and calculated using the same formula. However, the novel metric, which measures the novelty of generated compounds, has varied definitions across studies and has sometimes been excluded from performance evaluations. In the previous study [19], the Novel metric was defined as the fraction of molecules used in model training not present in the generated compound set. This refers to the novelty calculated from the perspective of the molecule set used for model training.

Therefore, we redefined this metric to evaluate the model’s ability to generate diverse compounds from the perspective of the generated molecule set. We defined Novel as the fraction of novel compounds among the generated valid compounds, where novel compounds refer to those that have never been seen during the training process. Accordingly, the count of these novel compounds is calculated by taking the set difference between the generated compound set and the molecule dataset used in training. When the actual molecular dataset used for training is defined as $X=\left\{x_{1},\ldots ,x_{h}\right\}$, where *h* is the number of the training dataset, the novel compound set *Z* is defined as $Z=\left\{p_{j}|p_{j}\notin X,1\leq j\leq b\right\}$. Thus, the Novel metric is calculated as follows:

(5) $$Novel=\frac{n(Z)}{n(P)}.$$

## 4. Conclusions

In this paper, we proposed an enhanced model for generating drug-like molecular compounds. To achieve the robust generation of valid compounds, we changed the representation of drug molecules to SELFIES and optimized the fine-tuning process. Specifically, we added an adapter module to our pre-trained model and fine-tuned it using a new training strategy. This strategy alternates between adapter module fine-tuning, which trains the parameters of the adapter module, and main module fine-tuning, which sequentially trains the remaining parameters.

The proposed Adapt-cMolGPT achieved a higher performance than two baseline models, cRNN and cMolGPT, on tasks with or without target proteins, as indicated by standard metrics. Particularly, by using SELFIES strings, our model exhibited exceptional robustness in the validity of target-specific drug generation. Additionally, by employing the new fine-tuning strategy, our model overcame the performance degradation issue in specialized tasks using small-scale datasets.

In summary, our Adapt-cMolGPT enhances drug-like molecular compound generation by utilizing SELFIES representation and an optimized fine-tuning process with an adapter module. It outperformed baseline models, cRNN and cMolGPT, particularly in target-specific drug generation, and effectively addressed performance degradation in specialized tasks with small-scale datasets. Therefore, the proposed method is expected to be widely used in the early stages of drug discovery for designing drugs targeting specific proteins.

## Figures and Tables

**Table 1:** Performance metrics for baseline models: fraction of valid molecules, fraction of unique molecules among 1000 and 10,000 valid molecules, and fraction of novel molecules derived from valid molecules.

Model	Valid	Unique@1k	Unique@10k	Novel
cMolGPT	0.985	1.0	**1.0**	0.835
Adapt-cMolGPT	**1.0**	**1.0**	0.999	**0.999**

The best value for each metric is in bold.

**Table 2:** Evaluation metrics: the fraction of valid molecules, the fraction of unique molecules among 10,000 valid molecules, and the fraction of novel molecules derived from valid molecules.

Target	Model	Valid	Unique@10k	Novel
EGFR	cRNN	0.926	0.862	0.946
	cMolGPT	0.842	0.922	0.955
	Adapt-cMolGPT	**1.0**	**0.944**	**1.0**
HTR1A	cRNN	0.922	0.845	0.782
	cMolGPT	0.885	0.889	0.889
	Adapt-cMolGPT	**1.0**	**0.962**	**1.0**
S1PR1	cRNN	0.921	0.861	0.947
	cMolGPT	0.905	0.869	0.912
	Adapt-cMolGPT	**1.0**	**0.931**	**1.0**

The best value for each metric is in bold.

**Table 3:** Percentage of molecules within the good drug-like property range for each target protein.

	MW ([200, 500])	TPSA ([20, 130])	LogP ([−1, 6])	HBD ([0, 5])	HBA ([0, 10])	QED ([0.4, 1])	SA ([1, 5])
EGFR	71.68%	93.95%	89.63%	99.20%	99.16%	51.66%	80.64%
HTR1A	89.30%	93.12%	97.51%	99.89%	99.92%	78.28%	87.91%
S1PR1	86.09%	89.50%	90.43%	99.39%	99.45%	55.41%	81.77%

## Data Availability

The datasets used in this study are available at https://github.com/VV123/cMolGPT (accessed on 14 May 2024).
